# 6,7-Diphenyl-5-thia-7-aza­spiro­[2.6]nonan-8-one 5,5-dioxide

**DOI:** 10.1107/S2414314623009379

**Published:** 2023-10-31

**Authors:** Hemant P. Yennawar, Michael W. Russell, Lee J. Silverberg

**Affiliations:** a Department of Biochemistry and Molecular Biology Pennsylvania State University, University Park, PA 16802, USA; b Pennsylvania State University, Schuylkill Campus, 200 University Drive, Schuylkill Haven, PA 17972, USA; University of Aberdeen, United Kingdom

**Keywords:** crystal structure, C—H⋯O, C—H⋯π, aromatic T-type stacking inter­actions, chair pucker

## Abstract

The racemic mixture of the title compound, C_19_H_19_NO_3_S, crystallizes in space group *P*  with two homochiral mol­ecules in each asymmetric unit. The seven-membered ring in both mol­ecules is in a pucker-chair conformation. The extended structure exhibits C—H⋯O hydrogen bonds, of which two connect crystallographically independent mol­ecules to generate a chain propagating along the *b-*axis direction. One C—H grouping of the cyclo­propyl ring is in close contact with the phenyl ring of the neighboring independent mol­ecule in C—H⋯π type inter­actions with carbon atom–ring-centroid distances of 3.544 (5) and 3.596 (4) Å. Other inter­actions are of the parallel–reciprocal type, with the chiral carbon atom of one mol­ecule donating a proton to an oxygen atom of the sulfone group of a symmetry-related mol­ecule and *vice-versa*. Symmetry-related mol­ecular pairs also exhibit T-type inter­actions between aromatic rings with inter­planar angles of 74.2 (2) and 69.2 (2)° and inter­centroid distances of 4.965 (4) and 5.114 (4) Å.

## Abstract 2

The seven-membered 1,3-thia­zepan-4-one ring system, like its six- and five-membered-ring analogues, is known to be biologically active and is of potential medicinal use. The crystal structure reported is of the sulfone of a 1,3-thia­zepan-4-one in which the seven-membered ring adopts a pucker-chair conformation.

## Graphical abstract

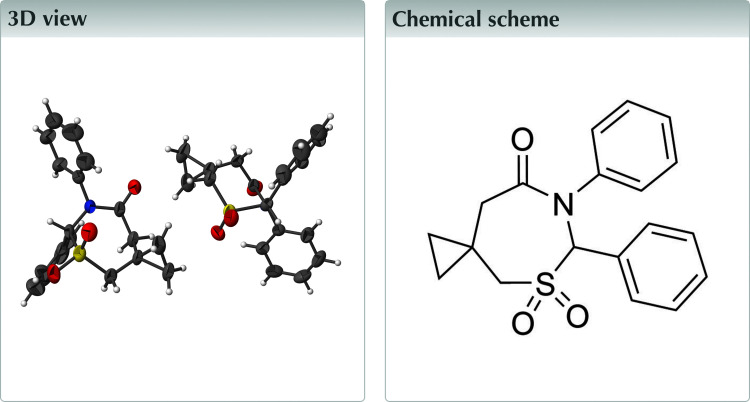

## Structure description

The seven-membered 1,3-thia­zepan-4-one ring system, like the similar six-membered 1,3-thia­zin-4-one and five-membered 1,3-thia­zolidin-4-one systems, is biologically active and of potential medicinal use. For example, the Bristol-Myers Squibb ACE/NEP inhibitor omapatrilat (C_19_H_24_N_2_O_4_S_2_) advanced to Phase II clinical trials (Graul *et al.*, 1999 ▸; Robl *et al.* 1997 ▸; Tabrizchi, 2001 ▸; Cozier *et al.*, 2018 ▸). Oxidation to the sulfone has been shown to change the biological activity of an isopenam 1,3-thia­zepan-4-one (Hwu *et al.*, 1999 ▸). *S*-Oxides of 1,3-thia­zin-4-ones have shown greater activity than the sulfides from which they were synthesized (Surrey *et al.*, 1958 ▸). Here we report the crystal structure of the sulfone derivative **1** (Silverberg, 2022 ▸) of 1,3-thia­zepan-4-one **2** (Yennawar & Silverberg, 2013 ▸). Although we have isolated the corresponding sulfoxide **3** (Silverberg, 2022 ▸), we have not been able to form it selectively and have not yet obtained a crystal structure.

**Figure 1:**
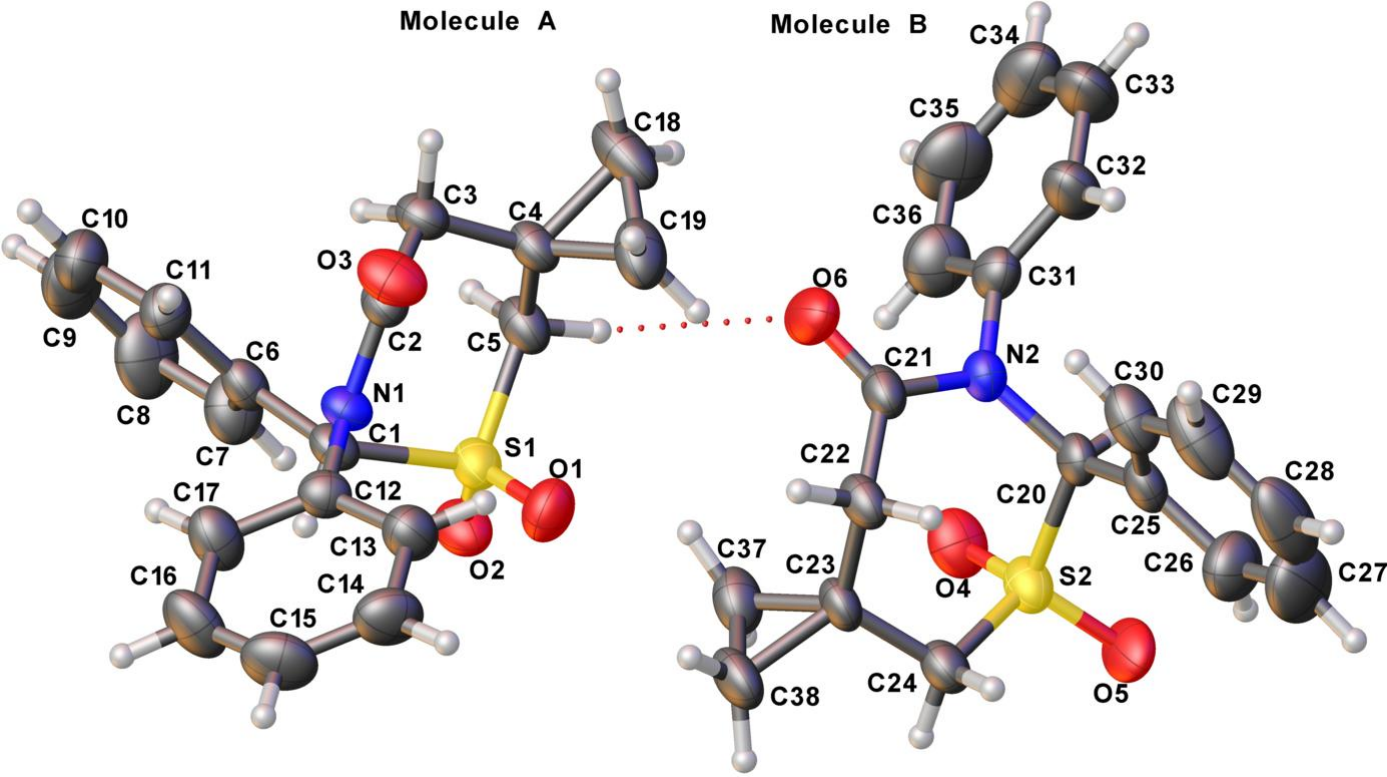
The mol­ecular structure of the title compound with displacement ellipsoids drawn at the 50% probability level. C—H⋯O inter­actions are shown as dashed lines.

The asymmetric unit of **1** is comprised of two independent mol­ecules (*A* containing C1 and *B* containing C20, Fig. 1 ▸), each consisting of a cyclo­propane ring, a pair of phenyl rings and a seven-membered heterocycle displaying a chair-pucker conformation in both mol­ecules. For the C1 mol­ecule, *q*(2) = 0.463 (4) Å, *q*(3) = 0.728 (3) Å, φ(2) = 92.7 (4)°, φ(3) = 336.2 (3)° and the total puckering amplitude *Q* = 0.863 (3) Å, with equivalent data of 0.444 (4) Å, 0.729 (3) Å, 90.2 (4)°, 335.4 (3)° and 0.853 (3) Å, respectively for the C20 mol­ecule. We reported similar puckering of the 1,3-thia­zepan-4-one ring previously (Yennawar *et al.*, 2019 ▸). The stereogenic centers (C1 and C20) in the arbitrarily chosen asymmetric unit both have *R* configurations but crystal symmetry generates a racemic mixture.

**Figure 2:**
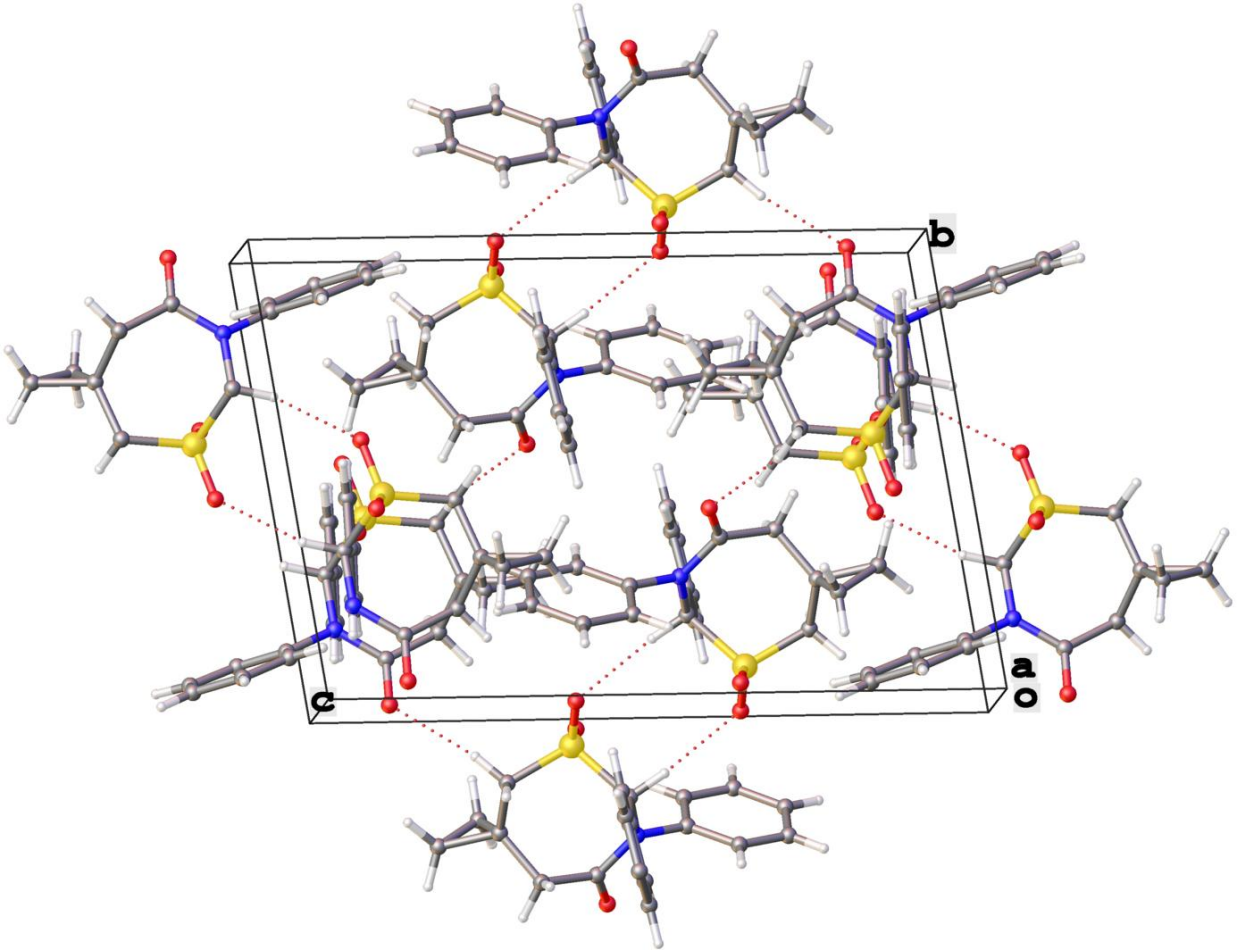
Packing diagram viewing down *a* axis, showing C—H⋯O hydrogen bonds between mol­ecules.

The packing of **1** is consolidated by a number of C—H⋯O and C—H⋯π type inter­actions (Fig. 2 ▸ and Table 1 ▸). One pair of C—H⋯O bonds, C5—H5*A*⋯O6 [C⋯O = 3.460 (5) Å, C—H⋯O = 161°] and C24—H24*A*⋯O3 [3.413 (5) Å and 165°], wherein the carbonyl oxygen atom of one mol­ecule accepts a C—H grouping of the heterocycle of another, form a chain of alternating crystallographically independent mol­ecules along the *b* axis direction. Independent neighbors along the [10 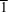 ] direction participate in C—H⋯π type inter­actions (Tsuzuki, 2000 ▸) wherein a C—H moiety (C18/C38) of the cyclo­propyl ring makes a close contact [C⋯π = 3.596 (5) and 3.544 (4) Å] with the centroid of an adjacent phenyl ring (C25–C30 and C6–C11, respectively]. Additionally, parallel *give-and-take* C—H⋯O inter­actions are seen between the symmetry-related pairs of mol­ecules wherein the chiral carbon atom (C1 and C20) of one donates a proton to one of the sulfone oxygen atoms (O2 and O5, respectively) on the heterocyclic ring of its neighbor in a reciprocal fashion.

## Synthesis and crystallization

6,7-Diphenyl-5-thia-7-aza­spiro­[2.6]nonan-8-one **2** (Yennawar & Silverberg, 2013 ▸) (0.0831 g, 0.267 mmol) was dissolved in glacial acetic acid (1.2 ml). An aqueous solution of KMnO_4_ (0.0853 g, 0.535 mmol in 1.45 ml water) was added dropwise at room temperature with vigorous stirring. The reaction was followed by TLC. Solid sodium bis­ulfite (NaHSO_3_/Na_2_S_2_0_5_) was added until the solution remained colorless. 1.45 ml of water were added and stirred for 10 min. The mixture was extracted with CH_2_Cl_2_ (3 × 5 ml). The organics were combined and washed once with sat. NaCl. The solution was dried over Na_2_SO_4_ and filtered. The product **1** was purified by chromatography in a silica gel microcolumn [0.0638 g, 70% yield. m.p. 186.6–187.7°C (decomposition)]. Crystals were grown by slow evaporation of an ethanol solution.

## Refinement

Crystal data, data collection and structure refinement details are summarized in Table 2 ▸.

## Supplementary Material

Crystal structure: contains datablock(s) I. DOI: 10.1107/S2414314623009379/hb4455sup1.cif

Structure factors: contains datablock(s) I. DOI: 10.1107/S2414314623009379/hb4455Isup2.hkl

Supporting information file. DOI: 10.1107/S2414314623009379/hb4455Isup3.mol

Supporting information file. DOI: 10.1107/S2414314623009379/hb4455Isup4.cml

CCDC reference: 2303670

Additional supporting information:  crystallographic information; 3D view; checkCIF report

## Figures and Tables

**Table 1:** Hydrogen-bond geometry (Å, °)

*D*—H⋯*A*	*D*—H	H⋯*A*	*D*⋯*A*	*D*—H⋯*A*
C1—H1⋯O2^i^	0.98	2.29	3.242 (4)	165
C5—H5*A*⋯O6	0.97	2.53	3.460 (5)	161
C20—H20⋯O5^ii^	0.98	2.35	3.310 (4)	166
C24—H24*A*⋯O3^iii^	0.97	2.47	3.413 (5)	165

Symmetry codes: (i) 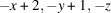 ; (ii) 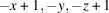 ; (iii) 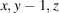 .

**Table 2:** Experimental details

Crystal data	
Chemical formula	C_19_H_19_NO_3_S
*M*_r_	341.41
Crystal system, space group	Triclinic, *P* 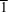
Temperature (K)	298
*a*, *b*, *c* (Å)	10.125 (5), 11.222 (5), 15.995 (7)
α, β, γ (°)	79.117 (8), 83.484 (9), 72.829 (8)
*V* (Å^3^)	1701.8 (13)
*Z*	4
Radiation type	Mo *K*α
μ (mm^−1^)	0.21
Crystal size (mm)	0.14 × 0.10 × 0.05

Data collection	
Diffractometer	Bruker *SMART* CCD
Absorption correction	Multi-scan (*SADABS*; Krause *et al.*, 2015 ▸)
*T*_min_, *T*_max_	0.698, 0.9
No. of measured, independent and observed [*I* > 2σ(*I*)] reflections	15037, 7728, 3674
*R*_int_	0.060
(sin θ/λ)_max_ (Å^−1^)	0.669

Refinement	
*R*[*F* ^2^ > 2σ(*F* ^2^)], *wR*(*F* ^2^), *S*	0.079, 0.207, 0.91
No. of reflections	7728
No. of parameters	433
H-atom treatment	H-atom parameters constrained
Δρ_max_, Δρ_min_ (e Å^−3^)	0.74, −0.45

Computer programs: *SMART* and *SAINT* (Bruker, 2001 ▸), *SHELXS* (Sheldrick, 2008 ▸), *SHELXL* (Sheldrick, 2015 ▸), and *OLEX2* (Dolomanov *et al.*, 2009 ▸).

## full crystallographic data

### Crystal data

**Table:**

C_19_H_19_NO_3_S	*Z* = 4
*M**_r_* = 341.41	*F*(000) = 720
Triclinic, *P*1	*D*_x_ = 1.332 Mg m^−^^3^
*a* = 10.125 (5) Å	Mo *K*α radiation, λ = 0.71073 Å
*b* = 11.222 (5) Å	Cell parameters from 1788 reflections
*c* = 15.995 (7) Å	θ = 2.4–25.1°
α = 79.117 (8)°	µ = 0.21 mm^−^^1^
β = 83.484 (9)°	*T* = 298 K
γ = 72.829 (8)°	Block, colorless
*V* = 1701.8 (13) Å^3^	0.14 × 0.10 × 0.05 mm

### Data collection

**Table:**

Bruker SMART CCD diffractometer	7728 independent reflections
Radiation source: fine-focus sealed tube	3674 reflections with *I* > 2σ(*I*)
Graphite monochromator	*R*_int_ = 0.060
Detector resolution: 8.34 pixels mm^-1^	θ_max_ = 28.4°, θ_min_ = 1.3°
phi and ω scans	*h* = −11→13
Absorption correction: multi-scan (SADABS; Krause *et al.*, 2015)	*k* = −14→15
*T*_min_ = 0.698, *T*_max_ = 0.9	*l* = −21→17
15037 measured reflections	

### Refinement

**Table:**

Refinement on *F*^2^	Primary atom site location: structure-invariant direct methods
Least-squares matrix: full	Secondary atom site location: difference Fourier map
*R*[*F*^2^ > 2σ(*F*^2^)] = 0.079	Hydrogen site location: inferred from neighbouring sites
*wR*(*F*^2^) = 0.207	H-atom parameters constrained
*S* = 0.91	*w* = 1/[σ^2^(*F*_o_^2^) + (0.1*P*)^2^] where *P* = (*F*_o_^2^ + 2*F*_c_^2^)/3
7728 reflections	(Δ/σ)_max_ < 0.001
433 parameters	Δρ_max_ = 0.74 e Å^−^^3^
0 restraints	Δρ_min_ = −0.45 e Å^−^^3^

### Special details

**Table:**

Experimental. The data collection nominally covered a full sphere of reciprocal space by a combination of 5 sets of ω scans each set at different φ and/or 2θ angles and each scan (30 s exposure) covering -0.300° degrees in ω. The crystal to detector distance was 5.82 cm.
Geometry. All esds (except the esd in the dihedral angle between two l.s. planes) are estimated using the full covariance matrix. The cell esds are taken into account individually in the estimation of esds in distances, angles and torsion angles; correlations between esds in cell parameters are only used when they are defined by crystal symmetry. An approximate (isotropic) treatment of cell esds is used for estimating esds involving l.s. planes.
Refinement. Refinement of F^2^ against ALL reflections. The weighted R-factor wR and goodness of fit S are based on F^2^, conventional R-factors R are based on F, with F set to zero for negative F^2^. The threshold expression of F^2^ > 2sigma(F^2^) is used only for calculating R-factors(gt) etc. and is not relevant to the choice of reflections for refinement. R-factors based on F^2^ are statistically about twice as large as those based on F, and R- factors based on ALL data will be even larger. The hydrogen atoms were placed geometrically (C—H = 0.93–0.98 Å) and refined as riding on their parent atoms with *U*_iso_(H) = 1.2*U*_eq_(C).

### Fractional atomic coordinates and isotropic or equivalent isotropic displacement parameters (Å^2^)

**Table:**

	*x*	*y*	*z*	*U*_iso_*/*U*_eq_	
S1	0.90079 (10)	0.56246 (8)	0.12862 (6)	0.0407 (3)	
S2	0.60160 (10)	0.07781 (8)	0.36592 (6)	0.0434 (3)	
O1	0.7581 (3)	0.5998 (2)	0.11179 (17)	0.0541 (7)	
O2	0.9835 (3)	0.4395 (2)	0.11397 (17)	0.0523 (7)	
O3	0.7584 (3)	0.9752 (2)	0.11388 (16)	0.0535 (7)	
O4	0.7454 (3)	0.0349 (2)	0.38132 (17)	0.0552 (8)	
O5	0.5248 (3)	−0.0146 (2)	0.38104 (18)	0.0598 (8)	
O6	0.7253 (3)	0.4160 (3)	0.38174 (17)	0.0608 (8)	
N1	0.8788 (3)	0.8038 (2)	0.05663 (18)	0.0350 (7)	
N2	0.6102 (3)	0.2742 (3)	0.43865 (18)	0.0376 (7)	
C1	0.9765 (3)	0.6790 (3)	0.0601 (2)	0.0359 (8)	
H1	0.9802	0.6593	0.0026	0.043*	
C2	0.8408 (4)	0.8715 (3)	0.1232 (2)	0.0376 (8)	
C3	0.8994 (4)	0.8121 (3)	0.2094 (2)	0.0410 (9)	
H3A	0.8795	0.8772	0.2449	0.049*	
H3B	0.9993	0.7805	0.2013	0.049*	
C4	0.8430 (4)	0.7046 (3)	0.2561 (2)	0.0404 (9)	
C5	0.9171 (4)	0.5762 (3)	0.2345 (2)	0.0460 (10)	
H5A	0.8808	0.5137	0.2728	0.055*	
H5B	1.0146	0.5575	0.2440	0.055*	
C6	1.1264 (3)	0.6682 (3)	0.0744 (2)	0.0385 (9)	
C7	1.2248 (4)	0.5528 (4)	0.0859 (3)	0.0578 (12)	
H7	1.1992	0.4790	0.0887	0.069*	
C8	1.3633 (4)	0.5460 (5)	0.0933 (3)	0.0738 (15)	
H8	1.4290	0.4676	0.1019	0.089*	
C9	1.4028 (5)	0.6529 (5)	0.0882 (3)	0.0714 (14)	
H9	1.4948	0.6479	0.0938	0.086*	
C10	1.3073 (4)	0.7666 (4)	0.0749 (3)	0.0608 (12)	
H10	1.3346	0.8398	0.0711	0.073*	
C11	1.1706 (4)	0.7764 (4)	0.0669 (2)	0.0474 (10)	
H11	1.1071	0.8558	0.0565	0.057*	
C12	0.8103 (4)	0.8537 (3)	−0.0220 (2)	0.0375 (9)	
C13	0.6711 (4)	0.8734 (4)	−0.0242 (3)	0.0497 (10)	
H13	0.6188	0.8561	0.0258	0.060*	
C14	0.6076 (4)	0.9188 (4)	−0.1002 (3)	0.0612 (12)	
H14	0.5128	0.9318	−0.1015	0.073*	
C15	0.6851 (5)	0.9450 (4)	−0.1743 (3)	0.0640 (13)	
H15	0.6424	0.9773	−0.2256	0.077*	
C16	0.8237 (5)	0.9233 (4)	−0.1722 (3)	0.0629 (12)	
H16	0.8766	0.9376	−0.2226	0.076*	
C17	0.8872 (4)	0.8802 (4)	−0.0959 (3)	0.0527 (11)	
H17	0.9817	0.8691	−0.0946	0.063*	
C18	0.7895 (5)	0.7086 (4)	0.3475 (3)	0.0662 (13)	
H18A	0.8042	0.6290	0.3865	0.079*	
H18B	0.7934	0.7796	0.3726	0.079*	
C19	0.6911 (4)	0.7337 (4)	0.2799 (3)	0.0598 (12)	
H19A	0.6348	0.8200	0.2640	0.072*	
H19B	0.6457	0.6694	0.2779	0.072*	
C20	0.5201 (3)	0.1954 (3)	0.4352 (2)	0.0378 (9)	
H20	0.5210	0.1453	0.4925	0.045*	
C21	0.6467 (4)	0.3572 (3)	0.3713 (2)	0.0422 (9)	
C22	0.5898 (4)	0.3741 (3)	0.2860 (2)	0.0427 (9)	
H22A	0.6076	0.4488	0.2507	0.051*	
H22B	0.4902	0.3889	0.2938	0.051*	
C23	0.6501 (4)	0.2623 (3)	0.2390 (2)	0.0398 (9)	
C24	0.5805 (4)	0.1577 (3)	0.2609 (2)	0.0443 (9)	
H24A	0.6180	0.0975	0.2221	0.053*	
H24B	0.4823	0.1933	0.2526	0.053*	
C25	0.3683 (4)	0.2620 (3)	0.4236 (2)	0.0408 (9)	
C26	0.2755 (4)	0.1958 (5)	0.4163 (3)	0.0689 (13)	
H26	0.3076	0.1095	0.4150	0.083*	
C27	0.1370 (5)	0.2559 (7)	0.4112 (4)	0.0916 (18)	
H27	0.0770	0.2102	0.4041	0.110*	
C28	0.0860 (5)	0.3791 (7)	0.4159 (3)	0.0851 (18)	
H28	−0.0087	0.4178	0.4135	0.102*	
C29	0.1746 (5)	0.4483 (5)	0.4245 (3)	0.0760 (15)	
H29	0.1402	0.5342	0.4271	0.091*	
C30	0.3149 (4)	0.3891 (4)	0.4292 (3)	0.0558 (11)	
H30	0.3743	0.4353	0.4362	0.067*	
C31	0.6767 (4)	0.2524 (3)	0.5174 (2)	0.0451 (10)	
C32	0.5996 (5)	0.2989 (5)	0.5868 (3)	0.0657 (13)	
H32	0.5071	0.3449	0.5820	0.079*	
C33	0.6601 (6)	0.2772 (6)	0.6635 (3)	0.0850 (16)	
H33	0.6089	0.3094	0.7103	0.102*	
C34	0.7943 (7)	0.2088 (6)	0.6703 (4)	0.0921 (18)	
H34	0.8343	0.1936	0.7222	0.111*	
C35	0.8720 (6)	0.1616 (5)	0.6019 (4)	0.0887 (17)	
H35	0.9642	0.1150	0.6072	0.106*	
C36	0.8122 (4)	0.1838 (4)	0.5243 (3)	0.0695 (13)	
H36	0.8641	0.1521	0.4774	0.083*	
C37	0.8030 (4)	0.2257 (4)	0.2163 (3)	0.0552 (11)	
H37A	0.8570	0.2756	0.2322	0.066*	
H37B	0.8505	0.1364	0.2190	0.066*	
C38	0.7043 (4)	0.2870 (4)	0.1474 (2)	0.0568 (11)	
H38A	0.6921	0.2347	0.1088	0.068*	
H38B	0.6985	0.3738	0.1219	0.068*	

### Atomic displacement parameters (Å^2^)

**Table:**

	*U*^11^	*U*^22^	*U*^33^	*U*^12^	*U*^13^	*U*^23^
S1	0.0446 (6)	0.0385 (5)	0.0399 (6)	−0.0138 (4)	0.0059 (4)	−0.0100 (4)
S2	0.0508 (6)	0.0347 (5)	0.0426 (6)	−0.0130 (4)	0.0093 (4)	−0.0068 (4)
O1	0.0448 (16)	0.0613 (17)	0.0607 (19)	−0.0246 (13)	−0.0016 (13)	−0.0066 (14)
O2	0.0698 (18)	0.0385 (14)	0.0496 (17)	−0.0175 (13)	0.0096 (14)	−0.0138 (12)
O3	0.0642 (18)	0.0405 (15)	0.0470 (17)	0.0023 (13)	−0.0024 (13)	−0.0137 (12)
O4	0.0458 (16)	0.0501 (16)	0.0580 (18)	−0.0007 (13)	0.0026 (13)	−0.0042 (13)
O5	0.086 (2)	0.0417 (15)	0.0595 (19)	−0.0386 (15)	0.0152 (15)	−0.0067 (13)
O6	0.083 (2)	0.0677 (19)	0.0467 (18)	−0.0488 (17)	0.0014 (15)	−0.0049 (14)
N1	0.0386 (16)	0.0349 (15)	0.0309 (17)	−0.0068 (13)	0.0009 (13)	−0.0114 (13)
N2	0.0402 (17)	0.0414 (16)	0.0325 (17)	−0.0170 (14)	0.0031 (13)	−0.0034 (13)
C1	0.0384 (19)	0.0368 (18)	0.030 (2)	−0.0057 (15)	0.0036 (15)	−0.0106 (15)
C2	0.041 (2)	0.0339 (19)	0.037 (2)	−0.0116 (16)	0.0045 (16)	−0.0075 (16)
C3	0.050 (2)	0.042 (2)	0.032 (2)	−0.0127 (17)	0.0017 (17)	−0.0114 (16)
C4	0.048 (2)	0.041 (2)	0.030 (2)	−0.0103 (17)	0.0073 (16)	−0.0085 (15)
C5	0.054 (2)	0.044 (2)	0.036 (2)	−0.0131 (18)	0.0092 (17)	−0.0057 (16)
C6	0.0347 (19)	0.047 (2)	0.030 (2)	−0.0073 (17)	0.0036 (15)	−0.0074 (16)
C7	0.043 (2)	0.051 (2)	0.069 (3)	−0.0045 (19)	0.002 (2)	−0.003 (2)
C8	0.037 (2)	0.078 (3)	0.087 (4)	0.002 (2)	−0.001 (2)	0.003 (3)
C9	0.041 (3)	0.106 (4)	0.067 (3)	−0.025 (3)	−0.008 (2)	−0.006 (3)
C10	0.051 (3)	0.077 (3)	0.062 (3)	−0.030 (2)	0.006 (2)	−0.016 (2)
C11	0.042 (2)	0.052 (2)	0.051 (3)	−0.0154 (19)	0.0060 (18)	−0.0138 (19)
C12	0.037 (2)	0.0368 (19)	0.037 (2)	−0.0070 (16)	−0.0044 (16)	−0.0085 (15)
C13	0.046 (2)	0.061 (3)	0.043 (2)	−0.0164 (19)	−0.0015 (19)	−0.0106 (19)
C14	0.051 (3)	0.075 (3)	0.061 (3)	−0.013 (2)	−0.017 (2)	−0.017 (2)
C15	0.072 (3)	0.069 (3)	0.046 (3)	−0.008 (2)	−0.023 (2)	−0.006 (2)
C16	0.065 (3)	0.078 (3)	0.034 (2)	−0.009 (2)	0.001 (2)	0.001 (2)
C17	0.043 (2)	0.065 (3)	0.043 (3)	−0.0073 (19)	0.0023 (19)	−0.004 (2)
C18	0.097 (4)	0.050 (2)	0.040 (3)	−0.011 (2)	0.021 (2)	−0.0082 (19)
C19	0.056 (3)	0.052 (2)	0.067 (3)	−0.015 (2)	0.023 (2)	−0.015 (2)
C20	0.042 (2)	0.042 (2)	0.031 (2)	−0.0195 (16)	0.0059 (16)	−0.0013 (15)
C21	0.050 (2)	0.041 (2)	0.037 (2)	−0.0187 (18)	0.0144 (18)	−0.0101 (16)
C22	0.055 (2)	0.038 (2)	0.033 (2)	−0.0156 (17)	0.0106 (17)	−0.0046 (16)
C23	0.046 (2)	0.044 (2)	0.028 (2)	−0.0161 (17)	0.0114 (16)	−0.0050 (15)
C24	0.050 (2)	0.047 (2)	0.035 (2)	−0.0156 (18)	0.0091 (17)	−0.0108 (17)
C25	0.039 (2)	0.053 (2)	0.027 (2)	−0.0134 (18)	0.0067 (15)	−0.0030 (16)
C26	0.047 (3)	0.085 (3)	0.080 (4)	−0.024 (2)	0.007 (2)	−0.023 (3)
C27	0.047 (3)	0.142 (6)	0.094 (4)	−0.036 (3)	0.002 (3)	−0.029 (4)
C28	0.041 (3)	0.140 (5)	0.053 (3)	−0.003 (3)	0.001 (2)	−0.002 (3)
C29	0.065 (3)	0.077 (3)	0.056 (3)	0.009 (3)	0.014 (2)	0.003 (2)
C30	0.050 (2)	0.057 (3)	0.047 (3)	−0.004 (2)	0.0110 (19)	−0.0030 (19)
C31	0.051 (2)	0.050 (2)	0.040 (2)	−0.0262 (19)	−0.0021 (19)	−0.0022 (18)
C32	0.063 (3)	0.103 (4)	0.039 (3)	−0.036 (3)	−0.001 (2)	−0.012 (2)
C33	0.092 (4)	0.142 (5)	0.040 (3)	−0.062 (4)	0.003 (3)	−0.019 (3)
C34	0.104 (5)	0.128 (5)	0.061 (4)	−0.069 (4)	−0.027 (4)	0.016 (3)
C35	0.067 (3)	0.108 (4)	0.090 (5)	−0.025 (3)	−0.035 (3)	0.004 (4)
C36	0.056 (3)	0.075 (3)	0.073 (4)	−0.014 (2)	−0.008 (2)	−0.006 (3)
C37	0.052 (2)	0.053 (2)	0.057 (3)	−0.016 (2)	0.018 (2)	−0.012 (2)
C38	0.077 (3)	0.057 (2)	0.036 (2)	−0.028 (2)	0.019 (2)	−0.0075 (19)

### Geometric parameters (Å, º)

**Table:**

S1—O1	1.423 (3)	C16—H16	0.9300
S1—O2	1.434 (3)	C16—C17	1.381 (5)
S1—C1	1.833 (4)	C17—H17	0.9300
S1—C5	1.759 (4)	C18—H18A	0.9700
S2—O4	1.426 (3)	C18—H18B	0.9700
S2—O5	1.441 (3)	C18—C19	1.486 (6)
S2—C20	1.830 (4)	C19—H19A	0.9700
S2—C24	1.752 (4)	C19—H19B	0.9700
O3—C2	1.209 (4)	C20—H20	0.9800
O6—C21	1.217 (4)	C20—C25	1.513 (5)
N1—C1	1.451 (4)	C21—C22	1.499 (5)
N1—C2	1.378 (4)	C22—H22A	0.9700
N1—C12	1.442 (4)	C22—H22B	0.9700
N2—C20	1.458 (4)	C22—C23	1.520 (5)
N2—C21	1.377 (4)	C23—C24	1.508 (5)
N2—C31	1.442 (5)	C23—C37	1.501 (5)
C1—H1	0.9800	C23—C38	1.509 (5)
C1—C6	1.526 (5)	C24—H24A	0.9700
C2—C3	1.517 (5)	C24—H24B	0.9700
C3—H3A	0.9700	C25—C26	1.385 (5)
C3—H3B	0.9700	C25—C30	1.384 (5)
C3—C4	1.520 (5)	C26—H26	0.9300
C4—C5	1.499 (5)	C26—C27	1.370 (6)
C4—C18	1.505 (5)	C27—H27	0.9300
C4—C19	1.495 (5)	C27—C28	1.338 (7)
C5—H5A	0.9700	C28—H28	0.9300
C5—H5B	0.9700	C28—C29	1.382 (7)
C6—C7	1.376 (5)	C29—H29	0.9300
C6—C11	1.394 (5)	C29—C30	1.383 (6)
C7—H7	0.9300	C30—H30	0.9300
C7—C8	1.399 (6)	C31—C32	1.376 (6)
C8—H8	0.9300	C31—C36	1.365 (5)
C8—C9	1.358 (6)	C32—H32	0.9300
C9—H9	0.9300	C32—C33	1.383 (6)
C9—C10	1.350 (6)	C33—H33	0.9300
C10—H10	0.9300	C33—C34	1.354 (7)
C10—C11	1.375 (5)	C34—H34	0.9300
C11—H11	0.9300	C34—C35	1.369 (8)
C12—C13	1.364 (5)	C35—H35	0.9300
C12—C17	1.374 (5)	C35—C36	1.391 (7)
C13—H13	0.9300	C36—H36	0.9300
C13—C14	1.379 (5)	C37—H37A	0.9700
C14—H14	0.9300	C37—H37B	0.9700
C14—C15	1.378 (6)	C37—C38	1.497 (6)
C15—H15	0.9300	C38—H38A	0.9700
C15—C16	1.356 (6)	C38—H38B	0.9700
			
O1—S1—O2	118.34 (17)	C19—C18—C4	60.0 (3)
O1—S1—C1	106.13 (15)	C19—C18—H18A	117.8
O1—S1—C5	109.40 (18)	C19—C18—H18B	117.8
O2—S1—C1	107.56 (16)	C4—C19—H19A	117.7
O2—S1—C5	108.30 (16)	C4—C19—H19B	117.7
C5—S1—C1	106.48 (17)	C18—C19—C4	60.7 (3)
O4—S2—O5	117.82 (16)	C18—C19—H19A	117.7
O4—S2—C20	106.93 (17)	C18—C19—H19B	117.7
O4—S2—C24	109.41 (17)	H19A—C19—H19B	114.8
O5—S2—C20	107.33 (17)	S2—C20—H20	104.0
O5—S2—C24	108.42 (18)	N2—C20—S2	110.3 (2)
C24—S2—C20	106.33 (17)	N2—C20—H20	104.0
C2—N1—C1	125.4 (3)	N2—C20—C25	117.1 (3)
C2—N1—C12	119.0 (3)	C25—C20—S2	115.8 (3)
C12—N1—C1	115.4 (3)	C25—C20—H20	104.0
C21—N2—C20	125.6 (3)	O6—C21—N2	119.5 (4)
C21—N2—C31	118.1 (3)	O6—C21—C22	120.5 (3)
C31—N2—C20	116.0 (3)	N2—C21—C22	120.0 (3)
S1—C1—H1	104.4	C21—C22—H22A	108.7
N1—C1—S1	109.4 (2)	C21—C22—H22B	108.7
N1—C1—H1	104.4	C21—C22—C23	114.2 (3)
N1—C1—C6	116.7 (3)	H22A—C22—H22B	107.6
C6—C1—S1	116.0 (2)	C23—C22—H22A	108.7
C6—C1—H1	104.4	C23—C22—H22B	108.7
O3—C2—N1	120.5 (3)	C24—C23—C22	115.7 (3)
O3—C2—C3	120.3 (3)	C24—C23—C38	115.9 (3)
N1—C2—C3	119.1 (3)	C37—C23—C22	118.0 (3)
C2—C3—H3A	108.7	C37—C23—C24	117.5 (3)
C2—C3—H3B	108.7	C37—C23—C38	59.7 (2)
C2—C3—C4	114.3 (3)	C38—C23—C22	118.5 (3)
H3A—C3—H3B	107.6	S2—C24—H24A	108.9
C4—C3—H3A	108.7	S2—C24—H24B	108.9
C4—C3—H3B	108.7	C23—C24—S2	113.3 (3)
C5—C4—C3	115.7 (3)	C23—C24—H24A	108.9
C5—C4—C18	116.0 (3)	C23—C24—H24B	108.9
C18—C4—C3	117.8 (3)	H24A—C24—H24B	107.7
C19—C4—C3	118.0 (3)	C26—C25—C20	121.3 (3)
C19—C4—C5	118.3 (3)	C30—C25—C20	120.8 (4)
C19—C4—C18	59.4 (3)	C30—C25—C26	117.5 (4)
S1—C5—H5A	108.9	C25—C26—H26	119.6
S1—C5—H5B	108.9	C27—C26—C25	120.8 (5)
C4—C5—S1	113.5 (3)	C27—C26—H26	119.6
C4—C5—H5A	108.9	C26—C27—H27	119.3
C4—C5—H5B	108.9	C28—C27—C26	121.4 (5)
H5A—C5—H5B	107.7	C28—C27—H27	119.3
C7—C6—C1	121.4 (4)	C27—C28—H28	120.2
C7—C6—C11	117.8 (4)	C27—C28—C29	119.7 (5)
C11—C6—C1	120.4 (3)	C29—C28—H28	120.2
C6—C7—H7	119.9	C28—C29—H29	120.2
C6—C7—C8	120.2 (4)	C28—C29—C30	119.5 (5)
C8—C7—H7	119.9	C30—C29—H29	120.2
C7—C8—H8	119.7	C25—C30—H30	119.5
C9—C8—C7	120.6 (4)	C29—C30—C25	121.0 (5)
C9—C8—H8	119.7	C29—C30—H30	119.5
C8—C9—H9	120.3	C32—C31—N2	118.6 (4)
C10—C9—C8	119.4 (4)	C36—C31—N2	121.0 (4)
C10—C9—H9	120.3	C36—C31—C32	120.4 (4)
C9—C10—H10	119.3	C31—C32—H32	120.1
C9—C10—C11	121.3 (4)	C31—C32—C33	119.7 (4)
C11—C10—H10	119.3	C33—C32—H32	120.1
C6—C11—H11	119.8	C32—C33—H33	120.1
C10—C11—C6	120.5 (4)	C34—C33—C32	119.8 (5)
C10—C11—H11	119.8	C34—C33—H33	120.1
C13—C12—N1	121.1 (3)	C33—C34—H34	119.5
C13—C12—C17	119.7 (3)	C33—C34—C35	121.0 (5)
C17—C12—N1	119.3 (3)	C35—C34—H34	119.5
C12—C13—H13	119.8	C34—C35—H35	120.2
C12—C13—C14	120.4 (4)	C34—C35—C36	119.6 (5)
C14—C13—H13	119.8	C36—C35—H35	120.2
C13—C14—H14	120.1	C31—C36—C35	119.5 (5)
C15—C14—C13	119.9 (4)	C31—C36—H36	120.3
C15—C14—H14	120.1	C35—C36—H36	120.3
C14—C15—H15	120.2	C23—C37—H37A	117.7
C16—C15—C14	119.7 (4)	C23—C37—H37B	117.7
C16—C15—H15	120.2	H37A—C37—H37B	114.8
C15—C16—H16	119.7	C38—C37—C23	60.4 (2)
C15—C16—C17	120.6 (4)	C38—C37—H37A	117.7
C17—C16—H16	119.7	C38—C37—H37B	117.7
C12—C17—C16	119.8 (4)	C23—C38—H38A	117.8
C12—C17—H17	120.1	C23—C38—H38B	117.8
C16—C17—H17	120.1	C37—C38—C23	59.9 (3)
C4—C18—H18A	117.8	C37—C38—H38A	117.8
C4—C18—H18B	117.8	C37—C38—H38B	117.8
H18A—C18—H18B	114.9	H38A—C38—H38B	114.9
			
S1—C1—C6—C7	43.2 (4)	C9—C10—C11—C6	1.5 (6)
S1—C1—C6—C11	−143.7 (3)	C11—C6—C7—C8	2.6 (6)
S2—C20—C25—C26	44.9 (4)	C12—N1—C1—S1	−105.8 (3)
S2—C20—C25—C30	−142.2 (3)	C12—N1—C1—C6	120.0 (3)
O1—S1—C1—N1	38.4 (3)	C12—N1—C2—O3	−5.1 (5)
O1—S1—C1—C6	173.0 (2)	C12—N1—C2—C3	172.5 (3)
O1—S1—C5—C4	−53.8 (3)	C12—C13—C14—C15	−0.3 (6)
O2—S1—C1—N1	166.0 (2)	C13—C12—C17—C16	−1.7 (6)
O2—S1—C1—C6	−59.4 (3)	C13—C14—C15—C16	1.4 (7)
O2—S1—C5—C4	175.9 (3)	C14—C15—C16—C17	−2.7 (7)
O3—C2—C3—C4	106.8 (4)	C15—C16—C17—C12	2.8 (7)
O4—S2—C20—N2	39.2 (3)	C17—C12—C13—C14	0.4 (6)
O4—S2—C20—C25	175.0 (2)	C18—C4—C5—S1	150.0 (3)
O4—S2—C24—C23	−53.5 (3)	C19—C4—C5—S1	82.4 (4)
O5—S2—C20—N2	166.5 (2)	C20—S2—C24—C23	61.7 (3)
O5—S2—C20—C25	−57.7 (3)	C20—N2—C21—O6	−178.5 (3)
O5—S2—C24—C23	176.8 (2)	C20—N2—C21—C22	1.4 (5)
O6—C21—C22—C23	108.0 (4)	C20—N2—C31—C32	−76.4 (4)
N1—C1—C6—C7	174.5 (3)	C20—N2—C31—C36	101.6 (4)
N1—C1—C6—C11	−12.5 (5)	C20—C25—C26—C27	176.0 (4)
N1—C2—C3—C4	−70.8 (4)	C20—C25—C30—C29	−175.5 (4)
N1—C12—C13—C14	−178.7 (3)	C21—N2—C20—S2	65.9 (4)
N1—C12—C17—C16	177.4 (3)	C21—N2—C20—C25	−69.3 (4)
N2—C20—C25—C26	177.6 (3)	C21—N2—C31—C32	109.7 (4)
N2—C20—C25—C30	−9.5 (5)	C21—N2—C31—C36	−72.2 (5)
N2—C21—C22—C23	−71.9 (4)	C21—C22—C23—C24	85.7 (4)
N2—C31—C32—C33	178.8 (4)	C21—C22—C23—C37	−61.1 (4)
N2—C31—C36—C35	−178.3 (4)	C21—C22—C23—C38	−129.9 (4)
C1—S1—C5—C4	60.5 (3)	C22—C23—C24—S2	−66.5 (4)
C1—N1—C2—O3	179.9 (3)	C22—C23—C37—C38	−108.4 (4)
C1—N1—C2—C3	−2.5 (5)	C22—C23—C38—C37	107.6 (4)
C1—N1—C12—C13	113.9 (4)	C24—S2—C20—N2	−77.6 (3)
C1—N1—C12—C17	−65.2 (4)	C24—S2—C20—C25	58.1 (3)
C1—C6—C7—C8	175.9 (4)	C24—C23—C37—C38	105.4 (4)
C1—C6—C11—C10	−176.3 (3)	C24—C23—C38—C37	−108.1 (4)
C2—N1—C1—S1	69.3 (4)	C25—C26—C27—C28	−2.4 (8)
C2—N1—C1—C6	−64.9 (4)	C26—C25—C30—C29	−2.3 (6)
C2—N1—C12—C13	−61.5 (5)	C26—C27—C28—C29	1.4 (8)
C2—N1—C12—C17	119.3 (4)	C27—C28—C29—C30	−0.9 (7)
C2—C3—C4—C5	86.5 (4)	C28—C29—C30—C25	1.4 (6)
C2—C3—C4—C18	−130.0 (4)	C30—C25—C26—C27	2.8 (6)
C2—C3—C4—C19	−61.9 (4)	C31—N2—C20—S2	−107.4 (3)
C3—C4—C5—S1	−65.9 (4)	C31—N2—C20—C25	117.4 (3)
C3—C4—C18—C19	107.7 (4)	C31—N2—C21—O6	−5.3 (5)
C3—C4—C19—C18	−107.4 (4)	C31—N2—C21—C22	174.6 (3)
C5—S1—C1—N1	−78.1 (3)	C31—C32—C33—C34	−0.9 (8)
C5—S1—C1—C6	56.5 (3)	C32—C31—C36—C35	−0.2 (6)
C5—C4—C18—C19	−108.8 (4)	C32—C33—C34—C35	0.7 (9)
C5—C4—C19—C18	105.1 (4)	C33—C34—C35—C36	−0.2 (9)
C6—C7—C8—C9	−0.9 (7)	C34—C35—C36—C31	0.0 (8)
C7—C6—C11—C10	−2.9 (6)	C36—C31—C32—C33	0.7 (7)
C7—C8—C9—C10	−0.7 (7)	C37—C23—C24—S2	80.5 (4)
C8—C9—C10—C11	0.4 (7)	C38—C23—C24—S2	148.2 (3)

### Hydrogen-bond geometry (Å, º)

**Table:**

*D*—H···*A*	*D*—H	H···*A*	*D*···*A*	*D*—H···*A*
C1—H1···O2^i^	0.98	2.29	3.242 (4)	165
C5—H5*A*···O6	0.97	2.53	3.460 (5)	161
C20—H20···O5^ii^	0.98	2.35	3.310 (4)	166
C24—H24*A*···O3^iii^	0.97	2.47	3.413 (5)	165

Symmetry codes: (i) −*x*+2, −*y*+1, −*z*; (ii) −*x*+1, −*y*, −*z*+1; (iii) *x*, *y*−1, *z*.
